# Comprehensive analysis of mutational signatures and corresponding driver genes in cervical cancer from Xinjiang

**DOI:** 10.1016/j.bbrep.2026.102591

**Published:** 2026-04-17

**Authors:** Chaoyang Chen, Wenbo Zhao, Min Guo, Wenling Wang, Jie Ma, Mayinuer Niyazi, Kaichun Zhu

**Affiliations:** a Xinjiang Dingju Medical Laboratory Co., Ltd., No. 181, Xicai Road, New Urban Area, Urumqi, Xinjiang, China; b Xinjiang Dingju Biotechnology Co., Ltd., No. 181, Xicai Road, New Urban Area, Urumqi, Xinjiang, China; c Department of Gynecology, People's Hospital of Xinjiang Uygur Autonomous Region, Urumqi, China; d Xinjiang Cervical Cancer Prevention and Treatment Clinical Research Center, China

**Keywords:** Cervical cancer, Somatic mutation, Mutational signatures, Driver gene, Signaling pathway

## Abstract

**Objective:**

To investigate the mutational characteristics of cervical cancer in Xinjiang and their relationships with tumor driver genes and affected signaling pathways.

**Methods:**

Twenty-nine pairs of cervical cancer tissues and matched peripheral blood samples were subjected to Whole Exome Sequencing. Somatic mutation sites and tumor mutational burden were identified, and core mutational signatures using non-negative matrix factorization (NMF). Driver genes were screened, and pathway enrichment analysis was performed to clarify the associations between core oncogenic pathways and driver genes.

**Results:**

A total of 10,076 nonsynonymous mutations in the coding region were identified. High-frequency mutated genes included members of the MUC family, HRNR, and NBPF1. Single-base substitutions were dominated by C > T (38.63%). Three core mutational signatures were identified: Signature A (undefined), Signature B (APOBEC-mediated deamination), and Signature C (endogenous damage/mismatch repair deficiency). Samples were divided into two groups based on these signatures: Group 1 (high C > G/C > T mutations) and Group 2 (high C > T mutations). Eleven driver genes were identified; among SLC24A1 was a driver gene in both the overall samples and subgroups, suggesting it is a key driver gene in cervical cancer. Gene mutations were mainly enriched in five pathways: NOTCH, RTK-RAS, WNT, Hippo and PI3K. Subgroup pathway signatures consistent with the overall cohort, differing only in mutation frequencies.

**Conclusion:**

This study clarified cervical cancer genomic heterogeneity, revealed core mechanisms (APOBEC activation and endogenous DNA damage), and confirmed SLC24A1 as key driver gene. It provides important genomic evidence for elucidating the pathogenesis of cervical cancer and exploring potential therapeutic targets.

## Highlights

• TMB in cervical lesions exceeds TCGA-CESC, confirming variable genomic instability.
• Signatures: B (82.15% similar to Sig.13), C (91.51% similar to Sig.1), A (unclear).
• SLC24A1 is stably expressed in some samples and serves as a key target.
• Xinjiang cervical cancer mutational signatures—precision treatment stratification.

## 1 Introduction

Over an individual's lifetime, normal human cells gradually accumulate somatic mutations resulting from spontaneous genomic alterations [1]. These acquired mutations play a central role in cancer initiation and progression, with cancer genomes typically harboring 10^3^ to 10^5^ somatic mutations [2]. Mutations arise from diverse biological processes, with each generating distinct, non-random patterns termed mutational signatures. These signatures reflect context-specific nucleotide preferences shaped by distinct etiological factors, rather than occurring randomly [3], and represent the cumulative mutational burden incurred during tumor development and progression [4]. Although most somatic mutations are non-driver (passenger) events, mutational signature analysis provides critical insights into historical mutagen exposures, DNA damage repair deficiencies, and genomic instability. This framework supports a mechanistic understanding of cancer and advances diagnostic and therapeutic approaches [5,6].

Previous large-scale analyses of somatic mutations across thousands of human cancers have defined more than 30 single-base substitution signatures and multiple genomic rearrangement signatures [7]. These signatures were systematically resolved using non-negative matrix factorization based on substitution type, flanking sequence context, mutational processes, and etiological origins. Thirty well-annotated signatures have been established, each linked to specific mutational processes and tumor etiologies [5,8]. For instance, *C > A/G > T* mutations predominate in the *TP53* gene of hepatocellular carcinoma associated with aflatoxin exposure [9]. Signature 3 is associated with impaired DNA double-strand break repair in *BRCA1/2*-mutant breast cancer [10]. Meanwhile, Signature 6 corresponds to defective DNA mismatch repair [11]. As the underlying causes of many signatures remain unclear, mutational signature analysis serves as a powerful tool to infer pathogenic drivers and stages of tumor evolution.

Among the vast number of somatic mutations, only a small subset act as drives mutations that promote by disrupting the balance between cell proliferation and apoptosis [12]. In cervical cancer, recurrent driver mutations in *PIK3CA*, *TP53*, *STK11*, *EP300*, *FBXW7*, and *HLA-B* have been identified as key contributors to malignant progression [13]. However, systematic studies focusing on somatic mutation patterns, mutational signature composition, and driver gene correlations in human papillomavirus (HPV)-positive cervical cancer patients in Xinjiang remain limited.

To address this gap, the present study enrolled HPV-positive cervical cancer patients from Xinjiang, adopting a paired design sample design of tumor tissue and peripheral blood. Whole Exome Sequencing (WES) was performed to achieve three objectives: First, to clarify the genomic patterns of somatic mutations and explore the correlation between mutation burden and tumor characteristics. Second, to characterize the composition of the mutational spectrum, including base substitution profiles, mutation proportions, and hotspot regions. Third, to identify associations between mutational signatures and driver genes, and clarify the regulatory effects of key mutations on signature formation. This study provides novel experimental evidence for the mutational evolutionary patterns of cervical cancer in Xinjiang. The findings will deepen the understanding tumorigenesis, support the development of precision diagnostic biomarkers and targeted therapies, and ultimately contribute to improved prognosis and quality of life for patients.

## 2 Materials and methods

### 2.1 Sample source

We collected data from cervical cancer patients who received treatment at the Department of Gynecology, People's Hospital of Xinjiang Uygur Autonomous Region, between 2017 and 2019. A total of 29 HPV-positive cervical cancer patients were enrolled after confirmation by HPV typing and pathological diagnosis. For each patient, we collected surgical tissue samples of cervical squamous cell carcinoma and paired peripheral blood samples. None of the patients had received radiotherapy, chemotherapy, undergone hysterectomy, or suffered from autoimmune diseases or immune deficiencies prior to sample collection. This study was approved by the hospital's Ethics Committee (Approval No.: KY2022080503), and written informed consent was obtained from all patients for sample collection and scientific testing.

### 2.2 Analysis of whole-exome sequencing data for somatic mutation detection

DNA was extracted and purified using the Magnetic Bead-based Extraction Kit (MGIEasy, 1000006988), and nucleic acid concentration was precisely quantified with the Qubit 3.0 Fluorometer (ThermoFisher, Q33216). Libraries were constructed using the MGIEasy DNA Library Preparation Kit for Enzymatic Fragmentation (MGIEasy, V2.0), and library quality was evaluated by DNA fragment size analysis with the Agilent 2100 Bioanalyzer (Agilent Technologies, G2939AA). Subsequently, libraries were re-quantified with the Qubit 3.0 Fluorometer, followed by whole-exome capture using the Agilent SureSelect Human All Exon V7 Kit. Samples were sequenced on the MGI 2000 platform with paired-end 2 × 150 bp reads.

For all 58 samples (29 tissue-blood pairs), reads were aligned to the human reference genome hg19 (GRCh37) using the Burrows-Wheeler Aligner (BWA, v0.7.17), and hg19 was chosen to ensure compatibility with existing annotation databases. To enhance analytical accuracy, raw sequencing data was filtered using Fastp (v0.20.0) to exclude reads with a Q20 quality score <90% and those with low mapping quality (MapQ <5). The Genome Analysis Toolkit (GATK, v4.1.8) was employed to improve mutation detection accuracy, which included local realignment around indel sites and base quality score recalibration. Using these recalibrated files, GATK's Mutect 2 module was utilized for mutation analysis by comparing paired blood and tumor samples, filtering out germline mutations to accurately identify somatic mutations in tumor tissues.

Additionally, the FilterMutectCalls module was applied to screen mutation sites, retaining those with a mutant allele frequency ≥0.05 and sequencing depth ≥10 × , while excluding sites with germline mutations (defined as a mutant allele frequency ≥0.01 in blood samples). This process yielded high-quality and reliable somatic mutation data for the tumors. Furthermore, the ANNOVAR software (v2.1.1) was used to annotate the chromosomal location of mutations, their positions within gene functional regions (e.g., exons, introns), and their potential impacts on protein structure (e.g., amino acid changes, the introduction of stop codons), thereby laying the foundation for subsequent functional analyses.

### 2.3 Analysis of tumor mutation burden and driver gene

Using the high-quality somatic mutation data obtained through screening, we performed analyses of tumor mutation burden (TMB) and driver genes. TMB was calculated as the number of non-synonymous somatic mutations (including missense, nonsense, frameshift, and splice site mutations) per million bases. Synonymous mutations and known germline polymorphic sites were excluded from TMB calculations.

Driver genes were identified using integrated bioinformatics approaches. MutSigCV (v1.4) was used to screen for genes with significantly higher mutation frequencies than expected (false discovery rate, FDR <0.05), while accounting for mutation frequency, gene length, mutation type, and background mutational patterns. OncodriveFML (v1.3) analyzed mutation clustering within gene functional domains to identify potential driver genes under positive selection. Final driver genes were required to meet two criteria: (1) mutations were detected in at least 2 samples; (2) they were identified as significantly mutated by at least one of the two algorithms.

### 2.4 Analysis of mutation patterns and mutational signatures

Somatic mutation site data from the 29 sample pairs were imported using the R package MutationalPatterns. A mutation matrix was constructed with the mut_matrix function, and six mutation types were generated with the mut_type_occurrences function. Flanking base information of each mutation site was obtained using the BSgenome package, yielding 96 trinucleotide change patterns (i.e., mutation patterns).

Non-negative matrix factorization (NMF) was calculated mutational signatures, with the optimal number of signatures determined by the inflection point of the cophenetic value. Signatures were extracted using the extract_signatures function. The identified signatures were compared to the 30 COSMIC database signatures (2015, https://cancer.sanger.ac.uk/signatures/signatures_v2/) via cluster analysis using cluster_signatures to verify their similarity. The 29 samples were clustered based on the inferred mutational signatures (cophenetic), and were grouped for subsequent analysis.

### 2.5 Enrichment analysis

Driver gene lists were standardized to ensure uniform gene nomenclature and then grouped according to the results of mutation signature clustering, generating group-specific driver gene sublists. To investigate the enrichment of these genes in Kyoto Encyclopedia of Genes and Genomes (KEGG) pathways, we employed the enrichKEGG function, with both p-value and q-value thresholds set to 0.05. Functions from the maftools package were used to visualize KEGG enrichment results, providing an intuitive representation of gene enrichment profiles across functional categories and pathways.

### 2.6 Statistical analysis

All statistical analysis were performed using GraphPad Prism 8.0. Descriptive statistics were used to summarize sequencing and mutation data, with quantitative variables presented as mean ± SD, median, and range. WES was performed on 29 pairs of cervical cancer tissues and peripheral blood samples to identify somatic mutations, and sequencing coverage depth was verified against the ≥30 × threshold to ensure reliable mutation detection. Nonsynonymous mutations in coding regions were screened, and their per-sample distribution was characterized. Localization analysis identified core genes enriched in mutations, and mutation rates were calculated to clarify functional associations. Subsequently, the distribution of TMB was described using mean ± SD and median. The Mann-Whitney *U* test was employed to statistically compare TMB differences between the SLC24A1 mutation cohort and the TCGA-CESC cohort. For categorical variables including age, ethnicity, and HPV genotype, group differences were assessed using the chi-square test or Fisher's exact test. A two-tailed P < 0.05 was defined as statistically significant.

## 3 Results

### 3.1 Somatic mutations and tumor mutational burden

This study adopted a paired-sample design, performing WES on cervical tissues and matched peripheral blood samples from 29 cervical cancer patients. In total, 14,737 somatic mutation sites were identified across the 29 sample pairs, with an average of 508.17 ± 447.12 mutations per sample. Sequencing coverage depth analysis revealed average depths of 66.54 × for cervical tissues and 64.52 × for peripheral blood samples. These values significantly exceeded the minimum ≥30 × threshold required for reliable somatic mutation detection, thereby substantially reducing the risk of false-negative results.

**Fig. 1:**
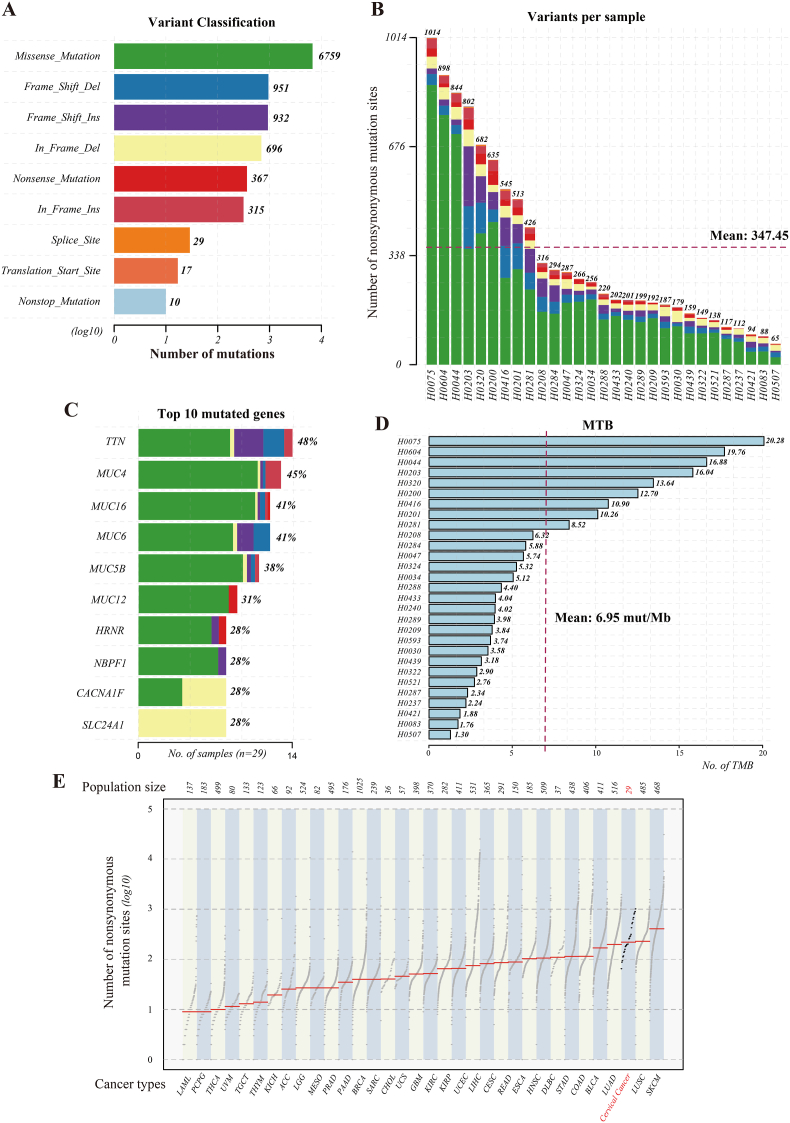
Somatic Mutation Profile and Mutation Burden Analysis. Note: A shows the statistical distribution of non-synonymous mutation sites and their types across the entire sample cohort; B presents the quantity and distribution of non-synonymous mutations in each individual sample; C displays the proportion of mutated genes in the samples as well as the top 10 mutated genes ranked by frequency; D illustrates the mutation burden of each sample, with the assessment based on non-synonymous mutation sites; and E provides a comparison of the number of mutation sites in this study with those of different cancer types in the COSMIC database. CESC, Cervical Squamous Cell Carcinoma.

Given that nonsynonymous mutations (including missense, nonsense, frameshift and splice site mutations) directly alter protein amino acid sequences, the study focused on nonsynonymous mutations in coding regions for in-depth analysis. The results showed that 10,076 nonsynonymous mutations were identified (Fig. 1A). Nonsynonymous mutations per sample exhibited a wide distribution, with a median of 220 (range: 65-1014) and a mean of 347.45 ± 273.19 (Fig. 1B). The distribution pattern was characterized by a median lower than the mean. This pattern suggested an enrichment of nonsynonymous mutation in some samples, which may reflect greater genomic instability.

Mutation localization analysis of frequently mutated gene demonstrated that these nonsynonymous mutations were mainly enriched in 10 core genes (Fig. 1C). Among these, the mucin (*MUC*) family genes showed for the highest mutation frequency. Mutations in *MUC* family genes and *HRNR* gene (mutation rate: 28%) were associated with impaired epithelial barrier function. Cell function-related genes *NBPF1*, *CACNA1F*, and *SLC24A1* (all with a 28% mutation rate) were linked to cell cycle or metabolic dysregulation.

To evaluate genomic instability in cervical cancer, we analyzed TMB values across all samples. The results showed that the median TMB of the 29 samples was 4.4 mutations per megabase (mut/Mb, range: 1.3-20.28 mut/Mb), with an mean of 6.95 ± 5.46 mut/Mb (Fig. 1D). The wide range of TMB values further confirmed the heterogeneity of genomic instability among cervical cancer samples.

To clarify the clinical representativeness of the mutation characteristics observed in this study, the median number of somatic mutations in our cohort was compared with that of the Cervical Squamous Cell Carcinoma (CESC) samples from The Cancer Genome Atlas (TCGA) database. The results indicated that the median number of somatic mutations in our cohort was significantly higher than that in the TCGA-CESC cohort (P < 0.05, Fig. 1E).

### 3.2 Association between somatic mutation patterns and mutation signatures

**Fig. 2:**
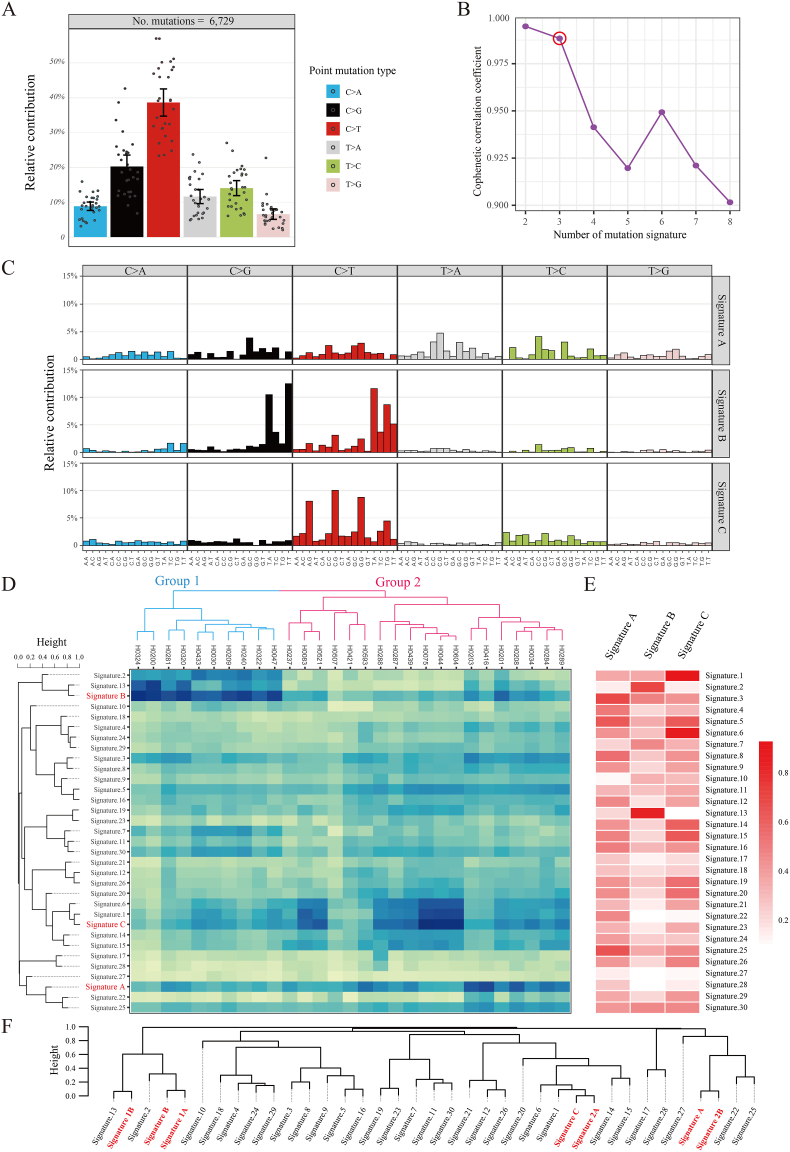
Analysis of Somatic Mutation Characteristics and Mutation Patterns in Samples. Note: A presents the statistics of six base substitution types in the samples; B describes the determination of the number of mutational signatures using the Non-negative Matrix Factorization (NMF) algorithm; C shows the distribution of samples across three distinct mutational signatures and their corresponding mutation patterns; D illustrates the clustering distribution of samples based on 33 mutational signatures; E displays a similarity heatmap comparing the 3 mutational signatures identified in this study with 30 mutational signatures from the COSMIC database; F outlines the grouping of samples via clustering based on 33 mutational signatures, followed by clustering and similarity analysis of the mutational signatures within each group.

To systematically characterize the molecular features of single-nucleotide mutation, the study identified 10,076 coding-region nonsynonymous mutations via WES. A total of 6729 high-quality sites were selected for in-depth analysis (Fig. 2A). Subsequent statistical analysis of the base substitution types of these single mutation sites revealed a significant preference for specific mutation types in cervical cancer. *C > T* mutations were most common, accounting for 38.63% ± 10.26%, followed by *C > G* at 20.26% ± 8.61%. The remaining four mutation types each contributed less than 15%. This finding indicates that cervical cancer mutations are mainly C-base-related, potentially associated with unique DNA damage mechanisms.

To unravel potential molecular mechanisms, the study employed the NMF algorithm. A matrix of 96 mutation patterns was constructed, based on one base each upstream and downstream of the mutation sites. This matrix was combined with the cophenetic correlation coefficient to identify three core mutation signatures: Signature A, Signature B, and Signature C (Fig. 2B). Further analysis of their distribution across the 96 mutation patterns (Fig. 2C) yielded key insights. Signature A exhibited a scattered distribution with no prominent mutation patterns and no clear associated mechanism. Signature B was highly concentrated in specific mutation patterns: *C > T* (*TCA*→*TTA*, *TCG*→*TTG*) or C > G (*TCA*→*TGA*, *TCT*→*TGT*). These patterns were commonly observed in the DNA cytosine deamination process mediated by *APOBEC* family proteins. Signature C was enriched in the *C > T* pattern (*ACG*→*ATG*, *CCG*→*CTG*, *GCG*→*GTG*), which is associated with endogenous DNA damage or DNA mismatch repair deficiency.

To delineate the biological mechanisms underlying these signatures, the study conducted similarity clustering analysis. The three identified signatures were compared with 30 known cancer mutation signatures from the COSMIC database (2015, v2) (Fig. 2D). The results (Fig. 2E) demonstrated clear similarity patterns. Signature A shared the highest similarity with COSMIC Signature 25 (64.09%) and Signature 22 (45.63%), but the similarity was too low to infer their potential mechanisms of action compared with known mutational signatures. Signature B showed high similarity with Signature 13 (82.15%) and Signature 2 (68.18%), which clearly confirmed its origin from the abnormal activation of *APOBEC* family proteins. Signature C was similar to Signature 1 (91.51%) and Signature 6 (86.46%), indicating that it involved mechanisms related to endogenous damage and mismatch repair abnormalities.

To explore inter-sample heterogeneity, the study combined the three identified signatures with 30 COSMIC signatures. Hierarchical clustering was then used to classify 29 samples into two clusters: Group 1 (10 samples, 34.48%) and Group 2 (19 samples, 65.52%). The NMF algorithm was applied to each cluster, with the optimal number of signatures determined by the cophenetic coefficient. This process extracted intra-cluster signatures (Fig. 2F). Key findings emerged from this analysis. Signature 1A and Signature 1B that were decomposed from Group 1 shared similarities of 92.11% and 93.46%, respectively, with the global Signature B. This suggests that mutations in this group 1 are primarily driven by APOBEC-related mechanisms. In contrast, Signature 2A and Signature 2B were decomposed from Group 2. Signature 2A showed 99.82% similarity to Signature C, while Signature 2B showed 92.93% similarity to Signature A. This indicated that mutations in this group 2 are associated with mechanisms such as endogenous damage and mismatch repair abnormalities.

Specifically, in terms of age distribution, a unified age cutoff (50 years) was adopted for consistent analysis. Neither Signature B (APOBEC-associated) nor the combined Signature A/C (linked to DNA mismatch repair and aging) showed significant enrichment in either younger or older patients (all P > 0.05). This indicates that the activity of both mutational signatures is not correlated with patient age. Regarding HPV genotype, no significant associations were detected between Signature B and high-risk HPV16/18 infection (P > 0.05), or between the combined Signature A/C and non-16/18 high-risk HPV infections (e.g., HPV52/58) (P > 0.05), suggesting that the mutational processes driven by these two signatures are not dependent on specific HPV genotypes. Similarly, with respect to ethnicity, neither Signature B nor the combined Signature A/C showed significant differences in activity between Han Chinese patients and ethnic minority patients (all P > 0.05), indicating that population-specific factors do not significantly affect the distribution of these two core mutational signatures (Table 1).

**Table 1:** Association between mutational signatures and clinical characteristics of patients.

Features	Group	Signature A/C (N = 19)	Signature B (N = 10)	P value
Age (50 years)	≥50	11	6	P = 0.91
	<50	8	4	
HPV Type	16/18	15	8	P = 0.69
	other	4	2	
Ethnicity	Uygur	14	6	P = 0.45
	Han	5	4	

### 3.3 Analysis of driver genes and enrichment of gene signaling pathways under different mutational signatures

To gain in-depth insights into the molecular regulatory mechanisms underlying tumor initiation and progression, and clarify the association between different mutational signatures and gene mutations as well as their impacts on tumor biological behaviors. Based on the sample clustering results previously obtained via mutational signature analysis, we used a combined analytical strategy using MutsigCV and Oncodriver. The complementarity of these two algorithms improves the accuracy and reliability of driver gene identification. Enrichment analysis was integrated to further validate the core pathways involved by the identified driver genes.

**Fig. 3:**
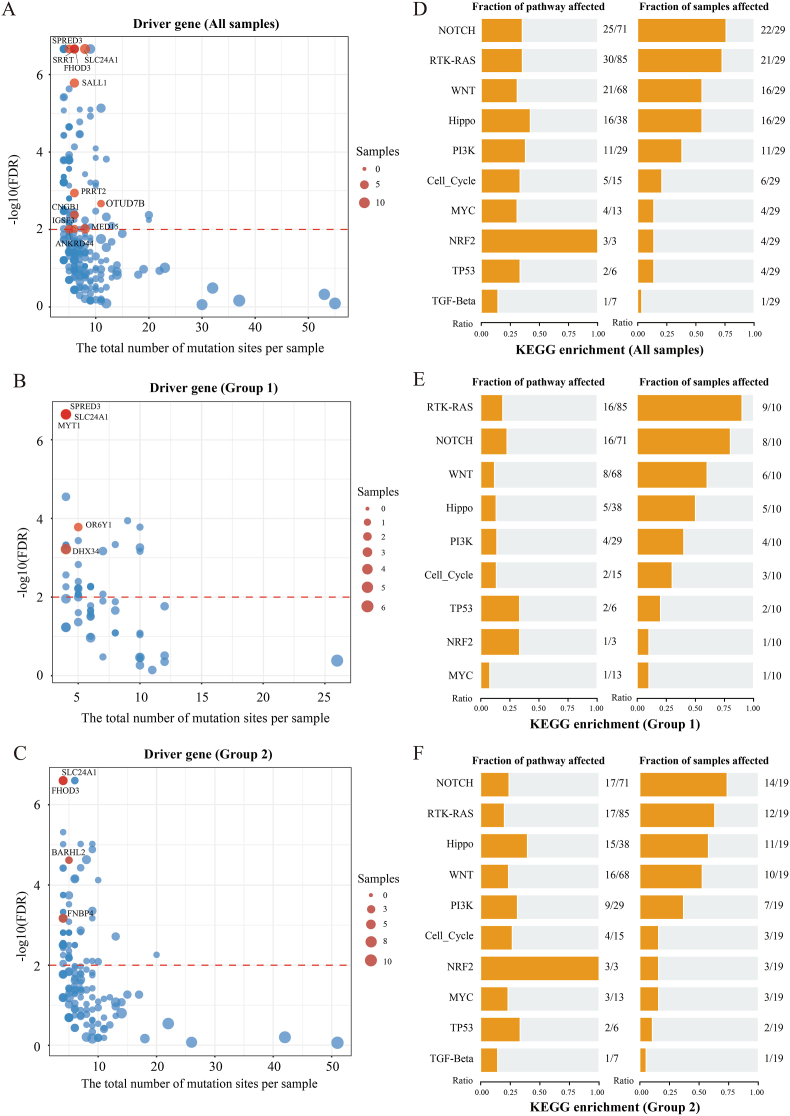
Analysis of Driver Genes and Gene-Enriched Signaling Pathways Based on Mutational Signatures. Note: A, B, and C show driver gene distributions in the total cohort, Group 1, and Group 2, respectively. Red dots indicate significant driver genes. D, E, and F show the corresponding enriched signaling pathways and their sample distributions. The left side represents the number of genes detected in each signaling pathway, and the right side represents the number of samples carrying these genes.

In the combined analysis of all cervical cancer samples, a total of 11 statistically significant (FDR <0.05) cervical cancer driver genes were identified. These mainly included *SLC24A1* (8/29, 27.59%), *MED15* (7/29, 24.14%), *FHOD3* (6/29, 20.69%), *SRRT* (6/29, 20.69%), *SALL1* (6/29, 20.69%), and five other genes (Fig. 3A). Subsequently, the entire sample set was divided into two subgroups based on mutational signatures. Group 1 (n = 10), which was dominated by high C > G and C > T mutations, and Group 2 (n = 19) was dominated by high C > T mutations. The driver genes of the two groups showed differences but overlapped in core genes. Driver genes in Group 1 mainly included *SLC24A1* (4/10, 40%), *SPRED3* (4/10, 40%), *DHX34* (4/10, 40%) (Fig. 3B). Driver genes in Group 2 mainly included *SLC24A1* (4/19, 21.05%), *FHOD3* (4/19, 21.05%), *FNBP4* (4/19, 21.05%) (Fig. 3C). Notably, *SLC24A1* was consistently present in the driver gene list across the entire sample set and both subgroups, with its mutation frequency ranking among the highest in each group. Importantly, all *SLC24A1* mutations in the mutated samples were deletions of entire codons in the reading frame, involving the deletion of the GAG sequence. This suggests that mutations in *SLC24A1* may play a crucial role in the initiation and progression of cervical cancer. To validate the driver genes identified in this study, we analyzed the public TCGA-CESC cervical cancer dataset, which yielded four driver genes: *PIK3CA*, *FBXW7*, *PTEN*, and *HLA-B*. These genes are entirely distinct from the 11 candidate driver genes detected in our study.

Given the consistent high mutation frequency and potential key role of *SLC24A1* in cervical cancer, we further performed comparative analyses to explore whether *SLC24A1* mutation status was associated with TMB and critical clinical covariates, including ethnicity, HPV genotype, and patient age at diagnosis. The results of these comparative analyses showed that none of the comparisons reached statistically significant levels (all P > 0.05). Specifically, there was no significant difference in TMB between SLC24A1-mutated and *SLC24A1* wild-type samples, indicating that the potential oncogenic effect of *SLC24A1* mutations is not associated with the overall tumor mutation load. Similarly, no significant correlations were observed between *SLC24A1* mutation status and ethnicity, HPV genotype, or patient age.

Combined with the results of KEGG pathway enrichment analysis, gene mutations in the entire sample set were mainly enriched in five core signaling pathways: NOTCH, RTK-RAS, WNT, Hippo, and PI3K. The mutation rates of these pathways were 75.86% (22/29), 72.41% (21/29), 55.17% (16/29), and 55.17% (16/29), and 37.93% (11/29), respectively (Fig. 3D). Further comparison of pathway enrichment characteristics between Group 1 and Group 2 revealed key findings. The signaling pathways enriched with major mutated genes in both subgroups were highly consistent with those in the entire sample set, with differences only in mutation rates. In Group 1, the mutation rates were 80% (8/10), 90% (9/10), 60% (6/10), 50% (5/10) and 40% (4/10), respectively (Fig. 3E). In Group 2, the corresponding mutation rates were 73.68% (14/19), 63.16% (12/19), 52.63% (10/19), 57.89% (11/19) and 36.84% (7/19), respectively (Fig. 3F). This result indicates that although the entire sample set and the two subgroups exhibit differences in mutational signatures, they share high similarity at the level of core oncogenic pathways.

## 4 Discussion

In the era of precision oncology, identifying driver genes is a core prerequisite for developing therapy development. For instance, hotspot driver genes such as EGFR in lung cancer [14] and KRAS in colorectal cancer [15] have enabled development of highly effective targeted agents. These drugs have achieved remarkable clinical benefits [16]. However, a common feature of tumors is the progressive accumulation of mutations [17]. Under the selective pressure of targeted therapies, the mutation spectrum of tumor cells gradually becomes more complex. Moreover, certain cancers (e.g., cervical cancer) lack well-defined early mutation patterns [18]. Cervical cancer has long been characterized by unclear driver gene obscurity. This condition is closely associated with persistent infection by high-risk HPV (HR-HPV). HR-HPV genome integration disrupts genomic stability, and random viral integration sites result in substantial interpatient mutational variability [19]. As a result, driver gene profiles often vary across study cohorts [20]. In the cervical cancer samples analyzed in this study, we detected significant differences in mutation counts among individual samples. This observation further supports the high inter-individual heterogeneity and biological complexity of cervical cancer.

To characterize the mutational patterns and heterogeneity of cervical cancer, we performed WES and extracted somatic mutation profiles. We identified three distinct mutational signatures, designated Signature A, Signature B, and Signature C. We then explored their biological mechanisms and clinical relevance by comparing them with the COSMIC (v2, 2015) mutational signature database. Among these, Signature A showed 64.09% similarity to Signature 25, observed in Hodgkin lymphoma [21] and 45.63% to Signature 22 observed in urothelial carcinoma [22]. Both signatures are linked to the induction of DNA double-strand breaks (DSBs) and generate *TCW* > *TAW* (*W=A/T*) mutations [23,24]. However, the similarity between Signature A and these known mutational signatures was too low to infer its potential mechanism of action. Signature B was highly similar to Signature 13 and Signature 2, with similarity rates of 82.15% and 68.18%, respectively. Both signatures are associated with the APOBEC cytidine deaminase family [25]. They often coexist [26] and have *TCW/CCW* (*W=A/T*) as their mutational core [25]. This feature is particularly prominent in cervical cancer [8]. Additionally, the HPV E6 protein can directly participate in the transcriptional upregulation of APOBEC3B, contributing to the formation of APOBEC3B-related mutational signatures [27]. This signature not only explains the high mutational burden of cervical cancer but also suggests that patients with this signature may respond better to immunotherapy. Signature C was highly consistent with Signature 1 and Signature 6, with consistency rates of 91.51% and 86.46%, respectively. These two signatures are present in almost all cancer types [11]. Signature 1 is age-related, while Signature 6 is associated with DNA mismatch repair (MMR) deficiency This result confirms that age is a key factor in endogenous mutations of cervical cancer and that some patients may have MMR deficiency.

In the overall cohort (29 paired samples), we identified a total of 11 candidate driver genes with significant mutation frequency (mutation rate >5%), including *SLC24A1*, *MED15*, *FHOD3*, *SRRT*, *SALL1*, *PRRT2*, *SPRED3*, *ANKRD44*, *CNGB1*, *IGSF3*, and *OTUD7B*. Among these, *SLC24A1* and *MED15* were the most frequently mutated driver genes, with *SLC24A1* mutations exhibiting a single mutation type: GAG codon deletion. Our 29 paired samples may have exhibit distinct demographic and clinical features compared with those in the TCGA-CESC dataset [28]. This discrepancy may be attributed to the inherent heterogeneity of cervical cancer and differences in sample characteristics, further underscoring the complexity of cervical cancer driver genes. Notably, the relatively small sample size of the present study (only 29 paired cases) represents a major limitation, which may reduce the statistical power for mutation frequency estimation and limit the generalizability of our findings.

Notably, relevant studies have reported that TMB is significantly associated with *FAT4* mutations in gastric cancer and have favorable prognostic value [29]. However, our study yielded different results regarding *SLC24A1* mutations. These findings suggest that the functional role of *SLC24A1* mutations in cervical cancer initiation and progression is unlikely to be confounded by variations in TMB, ethnic background, HPV infection type, or patient age, further supporting the independent potential of *SLC24A1* as a key driver gene in cervical cancer.

Based on these three mutational signatures, we further constructed a “mutational signature-driver gene-signaling pathway” framework. This framework is tightly linked to the five core cancer signaling pathways (NOTCH, RTK-RAS, WNT, Hippo, and PI3K) via 11 key driver genes. These 11 genes are significant associated with the five core pathways. *SPRED3* functions as a critical negative regulator of the RTK-RAS pathway [30]. Its mutations may lead to abnormal pathway activation, thereby promoting cervical cancer cell proliferation and invasion. *MED15*, *SALL1*, and *OTUD7B* regulate NOTCH and WNT signaling [[31], [32], [33]]. Their mutations may alter the transcriptional regulation of these pathways, thus disrupting the differentiation of cervical epithelial cells and the maintenance of stem cells. *FHOD3* and *IGSF3* are connected to the RTK/PI3K and Hippo pathways [34,35]. Their mutations may result in the loss of cell cycle control. *SLC24A1*, *SRRT*, and *ANKRD44* are primarily linked to PI3K signaling [36,37]. Their mutations may enhance cervical cancer cell survival and metastasis. In contrast, *CNGB1* and *PRRT2* exhibit relatively weak direct associations with these pathways. None of the mutation sites identified in these driver genes represent hotspot mutations.

Specifically, among Signature B-associated genes, *DHX34* (rs533574046, 3/10, 30%) may exert its effects by influencing mRNA metabolism or regulating relevant pathways [38]. Meanwhile, *MYT1* (rs765836332, 2/10, 20%; rs370664533, 2/10, 20%), which functions as a CDK1 inhibitory kinase that regulates the cell cycle [39], may act collectively in the context of the APOBEC mechanism. This therefore leads us to speculate that patients in this subgroup could potentially be more amenable to a combined treatment regimen of immunotherapy and APOBEC inhibitors. For Signature A- and C-related genes, *FNBP4* (rs759110657, 3/19, 15.79%) can lead to abnormal cell cycle progression and inactivation of the Hippo signaling pathway [40]. *BARHL2* (c.388_389delGA, 2/19, 10.53%; c.386_387insGGGG, 2/19, 10.53%), on the other hand, may influence the expression of WNT target genes by binding to T-cell factors [41], thereby indirectly regulating DNA damage repair efficiency. Driver genes in both subgroups involve DNA damage repair, reflecting genomic instability as a core carcinogenic mechanism and providing stratification for personalized treatment. Notably, *SLC24A1* (rs763504735, 8/29, 27.59%) is a PI3K-associated hub gene. It connects multiple mutational signatures through the regulation cellular calcium homeostasis [42].

Driver gene identification, subgroup enrichment analysis, and cross-validation in the full cohort revealed both significant differences and commonalities in driver genes. These results highlight shared molecular features of cervical cancer and subgroup-specific biological attributes, and enrich regional research on cervical cancer in Xinjiang. Meanwhile, three major patterns were identified First, Only a small subset of somatic mutations, such as *SPRED3* and *SLC24A1,* act as functional “driver mutations”. Second, Cancer-specific mutation signatures reflect etiology-mutagen-mutation relationships. For example, Signature B in cervical cancer is associated with HPV infection, and Signature 4 in lung cancer is related to smoking. Larger samples will help refine these associations. Third, Signature-driver gene correlation analysis supports a mechanism-target-treatment loop.

This study has several limitations, including a modest sample size, insufficient clinical follow-up data, and the lack of in vitro functional validation. Future research should adopt multi-center, large-cohort designs and integrated multi-omics approaches to address these constraints. Overall, this work represents the first characterization of mutation signatures in cervical cancer patients from Xinjiang. Stratification by these signatures and interrogation of subgroup-specific driver genes clarify the molecular basis of tumor heterogeneity. These findings facilitate the transition of cervical cancer care from conventional radiotherapy- and chemotherapy-based regimens to molecular subtype-guided precision therapy, providing a valuable research reference for future research and clinical translation.

## Ethics approval

We confirm that our study complies with all relevant national and international regulations governing research involving human subjects. We have taken all necessary steps to protect the privacy and confidentiality of the participants' information, ensuring that their identities remain anonymous in all published materials.

## Funding

Thanks for the support by Special Programme for Key Research and Development Project of Xinjiang Uygur Autonomous Region (Grant Number: 2022B03018-3).

## CRediT authorship contribution statement

**Chaoyang Chen:** Data curation, Software, Visualization, Writing – original draft. **Wenbo Zhao:** Project administration, Supervision, Validation. **Min Guo:** Conceptualization, Formal analysis, Investigation. **Wenling Wang:** Supervision, Validation, Writing – review & editing. **Jie Ma:** Supervision, Validation, Writing – review & editing. **Mayinuer Niyazi:** Conceptualization, Funding acquisition, Writing – review & editing. **Kaichun Zhu:** Conceptualization, Funding acquisition, Writing – review & editing.

## Declaration of competing interest

The authors declare that they have no known competing financial interests or personal relationships that could have appeared to influence the work reported in this paper.

## Data Availability

Data will be made available on request.
